# Seasonal exchange of microsporidian parasites between native and non-native pet-traded freshwater crustaceans: Is parasite spillover favored over spillback?

**DOI:** 10.1051/parasite/2025053

**Published:** 2025-09-25

**Authors:** Sebastian Prati, Andrea Carolina Reyes Camargo, Tom Jamonneau, Ilhan Ben Halima, Bernd Sures

**Affiliations:** 1 Department of Aquatic Ecology, University of Duisburg-Essen, Universitätsstr. 5 45141 Essen Germany; 2 Centre for Water and Environmental Research, University of Duisburg-Essen, Universitätsstr. 2 45141 Essen Germany; 3 Université de Montpellier, Place Eugène Bataillon 34095 Montpellier France; 4 Reserva: The Youth Land Trust P.O. Box 57277 Washington DC 20037 USA; 5 Association Guibétois 211 Chemin du triol 34343 Viols-le-Fort France; 6 Université de Toulouse (Paul Sabatier) 118 Route de Narbonne 31400 Toulouse France; 7 Research Center One Health Ruhr of the University Alliance Ruhr, University of Duisburg-Essen, Universitätsstr. 5 45141 Essen Germany.

**Keywords:** DNA barcoding, Freshwater ecosystems, Invasive non-native species, Microsporidia, *Neocaridina davidi*, Parasite transmission

## Abstract

The introduction of non-native pet-traded species poses potential threats to global biodiversity, particularly in freshwater ecosystems. This study investigated the seasonal dynamics of microsporidian infections in an established feral population of cherry shrimp (*Neocaridina davidi*) and the coexisting populations of crustaceans, comprising both native and non-native species, inhabiting the thermal waters of the Fontcaude Park and the nearby Mosson River in southern France. Our aim was to assess the potential occurrence of spillover and/or spillback events between *N. davidi* and co-occurring crustaceans, as well as the influence of seasonal dynamics on these interactions. The prevalence and diversity of microsporidian parasites exhibited strong seasonal variations. Although parasites associated with the pet trade were not detected, we highlight the acquisition of native parasites by feral *N. davidi*, which seems to be a suitable alternative host for native host-generalist microsporidians. Our findings indicate that all prerogatives for spillback events to occur are met. Feral *N. davidi* may establish and survive year-round in European rivers with natural thermal regimes. Thus, human-mediated introductions can potentially alter parasite transmission dynamics in these ecosystems.

## Abstract (fr)

L’introduction d’espèces non indigènes, commercialisées comme animaux de compagnie, constitue une menace potentielle pour la biodiversité mondiale, en particulier dans les écosystèmes d’eau douce. Cette étude examine la dynamique saisonnière des infections microsporidiennes au sein d’une population férale établie de crevettes de David (*Neocaridina davidi*) et des populations coexistantes de crustacés, indigènes et non indigènes, vivant dans les eaux thermales du parc de Fontcaude et de la Mosson, dans le sud de la France. Notre objectif était d’évaluer la survenue potentielle de débordement parasitaire et/ou de retransmission parasitaire entre *N. davidi* et les crustacés coexistants, ainsi que l’influence de la dynamique saisonnière sur ces interactions. La prévalence et la diversité des microsporidies parasites ont montré de fortes variations saisonnières. Bien que les parasites associés au commerce des animaux de compagnie n’aient pas été détectés, nous soulignons l’acquisition de parasites indigènes par *N. davidi* de la population férale, qui semble être un hôte alternatif approprié pour les microsporidies généralistes indigènes. Nos résultats indiquent que toutes les conditions sont réunies pour que des événements de retransmission se produisent. *Neocaridina davidi* pourrait s’établir et survivre toute l’année dans les rivières européennes à régimes thermiques naturels. Ainsi, les introductions d’origine humaine peuvent potentiellement modifier la dynamique de transmission des parasites dans ces écosystèmes.

## Introduction

The introduction of non-native pet-traded species is a growing phenomenon and a significant threat to global biodiversity [11, 15, 49, 58]. Once introduced, such species might be able to establish self-sustaining populations and eventually spread to other areas, becoming invasive, often asserting significant impacts on the local ecosystems [11, 15]. Freshwater ecosystems are especially affected by invasive species, which often lead to substantial biodiversity loss and major changes in energy flows and the physical environment [7, 15, 16, 21]. Direct impacts asserted by invasive species on receiving ecosystems typically involve biotic interactions such as competition, predation, hybridization, and pathogen transmission [7]. In contrast, indirect impacts often include changes in habitat structure, water clarity, organic matter, and nutrient concentrations [7]. Due to a complex interplay of a wide range of processes, including pathogen transmission and possible asymmetry in direct and indirect impact trajectories, the introduction of non-native pet-traded species with a known invasion history may lead to unpredictable outcomes.

The rise of the pet trade as a major pathway for the introduction of non-native species and their associated symbionts may enhance pathogen transmission between non-native and native species. This risk is exacerbated by insufficient biosecurity measures and potential immunosuppression of farmed pet animals, notably due to inbreeding and the relatively high density of individuals in aquaculture facilities, which can lead to higher pathogen prevalence compared to wild populations [38, 40, 49, 72]. For instance, ornamental cyprinids, such as *Carassius auratus* (Linnaeus, 1758) and *Cyprinus carpio* Linnaeus, 1758, are believed to be responsible for the spread of the anchor worm *Lernaea cyprinacea* Linnaeus, 1758, a crustacean parasite causing extensive hemorrhage and ulcerations in native Australian fish species [20]. Moreover, the introduction of non-native crayfish species of North American origin, including those linked to the pet trade, has led to severe declines and even extinctions of numerous native European crayfish species. A dire situation primarily caused by interspecific competition and the lack of immune defenses in native species against the crayfish plague, a deadly disease vectored by North American crayfish [43, 62]. As pathogens often exhibit broader environmental tolerances than their hosts [13], the introduction of non-native pet-traded species may facilitate the spread of pathogens beyond established climatic boundaries. Most pet-traded species originate from tropical or subtropical regions. They may struggle to establish themselves in colder climates, such as those found at higher latitudes (*e.g.*, Europe, Northeast Asia, or North America). Still, co-introduced pathogens might occasionally persist in novel ecosystems, even after their original host has disappeared [8, 58].

The “enemy release” theory suggests that non-native species partially lose their associated pathogens when introduced into new environments [65]. Pathogens may be lost due to handling, the subsampling effect (only uninfected individuals reach the transport stage), or during transport [11, 65]. After introduction, pathogens may fail to establish themselves because they either die shortly after, lack the ability to reproduce or have a reproduction rate too low to create a self-sustaining population [11]. Establishment can be especially challenging for non-native pathogens with complex life cycles, as they often depend on multiple specific hosts that may be absent in the receiving ecosystem [11, 65]. However, parasites and other pathogens with simple life cycles (*i.e.*, requiring only one host), particularly host-generalists, are more likely to persist in novel environments [53]. If the remaining pathogens are host-specialists, they may be constrained to the introduced non-native species and possibly a small number of closely related native host species; however, if they are host-generalists, spillover events to susceptible native species may occur. If multiple pathogens are introduced alongside an invasive host, a potential invasional meltdown, though rare, may occur [11]. Conversely, non-native species might also acquire local parasites due to a lack of immune defenses and/or close relatedness with native species, amplifying (spillback) or diluting (dilution) their prevalence in native populations [11, 29, 61, 63]. Thereafter, parasite transmission between non-native and native species is particularly relevant when dealing with potential pathogen reservoirs such as commercially successful freshwater pets. The latter, often immunosuppressed, may host generalist parasites with simple life cycles, and are more likely to be released into natural environments through aquarium dumping practices, enhancing the risk of pathogen spillover [17, 24, 43].

Popular pet-traded freshwater crustaceans such as crayfish (Astacidae) and shrimp (Atyidae) are known carriers of a wide range of pathogens and have established viable feral populations worldwide [4, 23, 40, 52]. One notable example is the commercially successful cherry shrimp *Neocaridina davidi* (Bouvier, 1904) (syn. *N. denticulata sinensis* and *N. heteropoda*), a freshwater species of Southeast Asian origin known for its high fecundity and environmental plasticity [52]. Feral self-sustaining *N. davidi* populations are currently found in Canada, Germany, France (metropolitan area and in the overseas territory of La Réunion), Hungary, Israel, Japan, Poland, Slovakia, and the USA (Hawaii) [24, 51, 52]. While invasive at lower latitudes, in continental Europe, *N. davidi* is thought to be limited to thermal waters, but recent evidence shows that, aided by a warming climate, it is expanding into colder waters [52]. Known ecological impacts of feral *N. davidi* include the replacement of native species with similar ecological niches [47], meiofaunal assemblage alterations [69], changes in the leaf-litter breakdown and thus energy flows in invaded areas [57], and possible parasite exchange with native and/or non-native species [52].

*Neocaridina davidi* hosts a wide range of commensals and parasites [3, 34, 40, 46, 52], some of which have already been co-introduced outside their native range [26, 39, 45, 52, 56]. Among them is the ecologically and economically relevant microsporidian parasite *Ecytonucleospora hepatopenaei* (Tourtip *et al.*, 2009) Wang *et al.*, 2023 (syn: *Enterocytozoon hepatopenaei*), which has been detected in pet-traded and feral *N. davidi* populations across Europe [52, 56]. This host-generalist parasite with a simple life cycle infects many freshwater, brackish, and marine invertebrates, often causing substantial economic losses in the shrimp aquaculture industry [10, 25, 27, 32, 44]. Recent evidence suggests that microsporidian parasites may be shared between feral *N. davidi* and the invasive Red Swamp crayfish (*Procambarus clarkii* (Girard, 1852) [52]. However, it is unclear whether spillover or spillback events may occur between feral *N. davidi* and native species in invaded ecosystems. An attempt to experimentally infect native amphipods and isopods using *N. davidi* tissues containing *E. hepatopenaei* spores was not successful [52]. Still, feral *N. davidi* can host microsporidian parasites phylogenetically close to those found in insects such as coleopterans and lepidopterans, suggesting that spillback events might occur [51]. Yet, parasitological investigations comparing the parasite communities of feral *N. davidi* with those of native species sharing the same habitat are scarce and consist only of snapshots, thereby not accounting for potential seasonal dynamics [52]. As with other parasites, microsporidian infections can display pronounced seasonality [50], potentially leading to undetected spillover or spillback events in studies with suboptimal sampling periods.

Hence, we decided to investigate the microsporidian fauna of a recently discovered feral population of *N. davidi* inhabiting the warm waters of the Fontcaude thermal park of Juvignac (Southern metropolitan France) since at least 2021 [24]. Its water hosts both native species, such as isopods and amphipods, which are known hosts for a wide range of microsporidian parasites, many of which are host generalists [18, 53], as well as fish and crustacean species of pet-traded origin [24]. The thermal water originating from a spring feeds into a pond and then outflows in a ~ 70 m long channel, gradually losing heat before being redirected to the nearby Mosson River *via* an underground pipe and/or surface overflow channels in the downstream section [24]. These settings provide an ideal environment for microsporidian parasite exchanges between native and non-native species, and eventually, the spread of propagules in the Mosson River.

Profiting from this unique opportunity, the current study aimed to assess whether microsporidian spillover and/or spillback events may have occurred between *N. davidi* and other native and non-native crustaceans living in the park and the nearby Mosson River, and whether seasonality influenced these events. To this end, we employed DNA barcoding to molecularly characterize native and non-native crustaceans and their microsporidian parasites across different seasons. Additionally, pet-traded shrimps and their parasites were DNA-barcoded to facilitate direct comparison with feral individuals. We first hypothesized that microsporidian parasites found in pet-traded *N. davidi* would be detected in the feral population, as observed elsewhere in Europe [51, 52, 56]. Second, we hypothesized that spillback would be more favored than spillover events due to the higher diversity and prevalence of generalist parasites in native species compared to *N. davidi* and a limited subset of introduced animals, possibly stemming from uninfected pet-traded individuals. Last, we hypothesized that the prevalence and diversity of microsporidians in both native and non-native species would vary between seasons.

## Material and methods

### Sampling and processing

Between March and November 2024, *N. davidi* individuals and co-occurring amphipods, decapods, and isopods were collected using hand nets and baited bottles in the Fontcaude thermal park of Juvignac (Hérault, France; 43.627707, 3.811910) following the French fishing act and a prefectural authorization (id: DDTM34-2024-05-14915). At this site, warm water originating from a thermal spring-fed pond flows through a channel toward the Mosson River, gradually cooling along its course. This environment supports the coexistence of native and non-native species, including several taxa introduced *via* the pet trade, such as *N. davidi* and the guppy, *Poecilia reticulata* Peters, 1859 [24]. *Neocaridina davidi* was first recorded in the thermal park in December 2023 and has maintained a stable population since then. Its presence, however, is believed to date back at least to 2021 [24]. Individuals of *N. davidi* have been observed year-round in both the warmer and colder areas of the water body, suggesting that, if able to reach the nearby Mosson River, they might survive in it. However, their presence in the Mosson River had not been previously investigated; thus, in September and November 2024, we sampled a section of the river spanning 500 m upstream and downstream of the thermal park channel outlet, using baited bottles in accordance with the French fishing act. Unfortunately, most of the samples collected in the Mosson in September were later stolen, along with part of the equipment. Fifty-two individuals of *N. davidi* were also purchased from local pet shops and private sellers, as well as one of Europe’s largest and most popular online stores in Germany. All crustaceans employed in the study were euthanized using eugenol [9], and upon death, they were fixed in 96% ethanol for molecular analyses of both hosts and parasites. All hosts were measured and dissected at the Aquatic Ecology laboratory of the University of Duisburg-Essen (Germany). The processing of samples included visual screening for internal parasites, gut removal to prevent microsporidian contamination, and the dissection of a small portion of muscle and hepatopancreatic tissue for downstream molecular analyses. Epibionts were not analyzed as their conservation in ethanol-preserved specimens is often poor [52].

### Host DNA extraction and amplification

We isolated DNA from hosts using a modified salt precipitation protocol [19]. Host molecular identification was performed using the universal eukaryotic primers LCO1490 (5′–GGTCAACAAATCATAAAGATATTGG–3′) and HCO2198 (5′–TAAACTTCAGGGTGACCAAAAAATCA–3′) [14], targeting the cytochrome oxidase I (COI) region. This primer pair has previously been used to amplify a wide range of crustaceans, including those analyzed in this study [52]. Additionally, we developed novel primers for *N. davidi*, targeting the mitochondrial 12S and 16S regions, based on published mitogenome sequences (NCBI GenBank accession numbers MN418055, NC_043865, and MK907783), to enhance the availability of alternative markers for future phylogenetic analyses. These were the following: 12SNeoF (5′–AAAGTGCGGGTTAAGATTGTGC–3′) and 12SNeoR (5′–TCCAGCACACCTACCTTGTTAC–3′), 16SNeoF (5′–TTGGCATCTCGAAGTGGAATGA–3′) and 16SNeoR. PCR reactions for hosts consisted of 20 μL assay with 10 μL of Dream-TaqTM Hot Start Green PCR Master Mix (Thermo Fisher Scientific, Waltham, MA, USA), 1.6 μL (5 μM) of each primer, 4.8 μL of nuclease-free water, and 2 μL of DNA template per reaction. PCR settings used for the primer pairs LCO1490–HCO2198 followed those used by [51], while those used for the newly developed primers targeting the 12S and 16S regions were as follows: initial denaturation for 2 min at 95 °C, followed by 30 cycles of 30 s denaturation at 95 °C, 40 s annealing at 59 °C and 90 s elongation at 72 °C, with a final elongation of 8 min at 72 °C.

### Microsporidian DNA amplification

Microsporidian parasites were identified using the universal primers V1F (5′–CACCAGGTTGATTCTGCCTGAC–3′) [74] and 1342R (5′–ACGGGCGGTGTGTACAAAGAACAG–3′) [41], targeting the small subunit ribosomal RNA gene (SSU rRNA). PCR reactions for microsporidians consisted of 20 μL composed of 10 μL of 2× AccuStart II PCR ToughMix (Quantabio, Beverly, MA, USA), 1 μL of each primer (0.5 μM), 0.35 μL of 50× GelTrack Loading Dye (Quantabio), 6.65 μL MilliQ water, and 1 μL of DNA template. PCR settings used for the primer pairs V1F-1342R followed those used by [51].

### Sequences and phylogenetic analyses

PCR products from hosts and microsporidians were sent unpurified to Microsynth Seqlab (Göttingen, Germany) for Sanger sequencing using LCO1490, 12SneoF, 16SneoF, and V1 primers, respectively. Raw sequences were quality-checked and edited using Geneious v2025.0.2 (GraphPad Software LLC d.b.a Geneious) and compared against GenBank records using BLASTN (https://blast.ncbi.nlm.nih.gov/). Host and parasite sequences were aligned using the MAFFT v7.490 algorithm with standard settings [28]. Maximum likelihood phylogenetic trees with bootstrap support values (1,000 replicates) were produced in IQ-Tree v2.4.0 [42] using the HKY + F + I substitution model for crustaceans and the TIM3 + F+I + R4 substitution model for microsporidians, both based on Bayesian Information Criterion scores. The respective outgroups were *Neocaridina palmata* (Shen, 1948) (MN701612) and *Metchnikovella dogieli* (Paskerova *et al.*, 2016) (MT969020). The names and circumscriptions of *N. davidi* clades followed those proposed by Prati *et al.* [52], while those of microsporidian orders followed Bojko *et al.* [5]. Following the naming used in previous works [50–56], we used the artificial name “Microsporidium sp.” for all microsporidian isolates lacking a formal description. The only exception was for undescribed isolates belonging to the genus *Nosema*, for which we referred to the most recent revision [2]. A haplotype network for *N. davidi* only was inferred using the Minimum Spanning Network method in PopArt v1.7 [33]. All the sequences generated in this study were submitted to NCBI GenBank [hosts: PV394804–PV394813 (COI), PV697566–PV697569 (12S), PV696985–PV696986 (16S); microsporidians: PV383432–PV383436 (SSU rRNA)].

### Statistical analyses

Sex, season, size, and origin of the shrimp individuals might influence parasite infections in *N. davidi*. However, direct comparisons between these variables were not feasible using a global Generalized Linear Model, as samples were collected in different months with unbalanced sampling effort. Thus, we first compared the sex ratios of sexed *N. davidi* individuals from different origins (Fontcaude Thermal Park, Mosson River, and pet trade) and separately examined differences across sampling months (March, September, October, and November) using a Chi-square (χ^2^) test of independence. Then, we compared microsporidian infections between feral and pet traded individuals using Fisher’s exact test. Lastly, we assessed whether the size of sexed individuals influenced microsporidian infections using a logistic regression with infected/uninfected as the dependent variable, and carapax size and sex (female/male) as independent explanatory variables. The results were reported as odds ratios (OR). Statistical and descriptive analyses were performed using the open-source software R v4.4.1 (R Core Team) through the RStudio graphical user interface (version 2024.12.1, Posit Software PBC).

## Results

### Sample collection

A large number of individuals of *N. davidi* were observed in the Fontcaude Thermal Park in all sampling months (March, September, and November). Likewise, we confirmed widespread presence of *N. davidi* along the investigated stretch of the Mosson River in September and November 2024, albeit in smaller numbers than observed in the park. The water parameters measured along the thermal gradient of the Fontcaude Thermal Park at the time of collection were 15.20–23.30 °C, 6.86–7.50 pH, and 3.88–7.53 mg/L DO in March to 21.50–24.00 °C, 7.20–7.66 pH, and 3.37–7.05 mg/L DO in September, and those in the nearby Mosson from 20.30 °C, 7.43 pH, 6.32 mg/L DO in September to 13.80 °C, 7.92 pH, and 9.70 mg/L DO in November.

In total, we collected and examined 320 *N. davidi* individuals (201 from the thermal park, 67 from the Mosson, and 52 from the pet trade). Field-collected *N. davidi* comprised 121 females, of which 34 were ovigerous, 125 males, and 22 immature individuals, while pet-traded ones included 24 females, none of which were ovigerous, 22 males, and six immature individuals. Two ovigerous females were detected in the Mosson between September and November 2024. Likewise, immature individuals (carapax size ranging from 1.55 to 2.97 mm) were detected in the Mosson River in both months. No difference in sex ratios was detected among *N. davidi* individuals from the thermal park, the Mosson, the pet trade, or across sampling months (χ^2^, all *p* > 0.28).

Additionally, we collected 126 co-occurring crustaceans from the thermal park and the Mosson which were later molecularly identified as *Asellus aquaticus* (Linnaeus, 1758) (*n* = 18, 99.84% similarity and 100% coverage to AY531783), *Atyaephyra desmarestii* (Millet, 1831) (*n* = 44, 98.91% similarity and 100% coverage to JX853920), *Gammarus pulex* (Linnaeus, 1758) (*n* = 53, 99.15% similarity and 93% coverage to PQ817982), and *P. clarkii* (*n* = 11, 100% similarity and coverage to JX441375). These were less widespread than *N. davidi,* as indicated by visual observations and the number of individuals collected.

### Phylogenetic relationships of *N. davidi*

**Figure 1:**
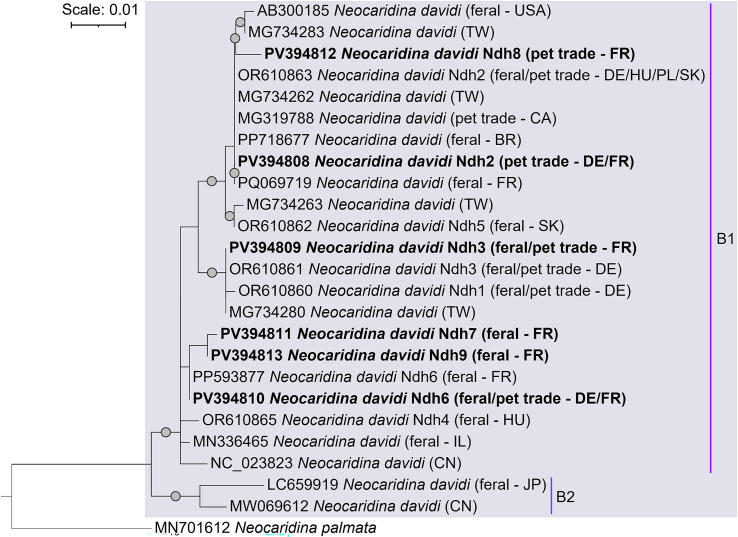
Maximum likelihood phylogenetic tree of *Neocaridina davidi* identified in this study. Grey dots represent bootstrap support values above 90%. Sequences obtained in this study are indicated in bold. The substitution model used was HKY + F + I, and *N. palmata* represents the outgroup. The names and circumscriptions of *N. davidi* clades followed [52]. Abbreviations: Brazil (BR), Canada (CA), China (CN), Germany (DE), France (FR), Hungary (HU), Israel (IL), Japan (JP), Slovakia (SK), Poland (PL), Taiwan (TW), and the United States of America (USA).

**Figure 2:**
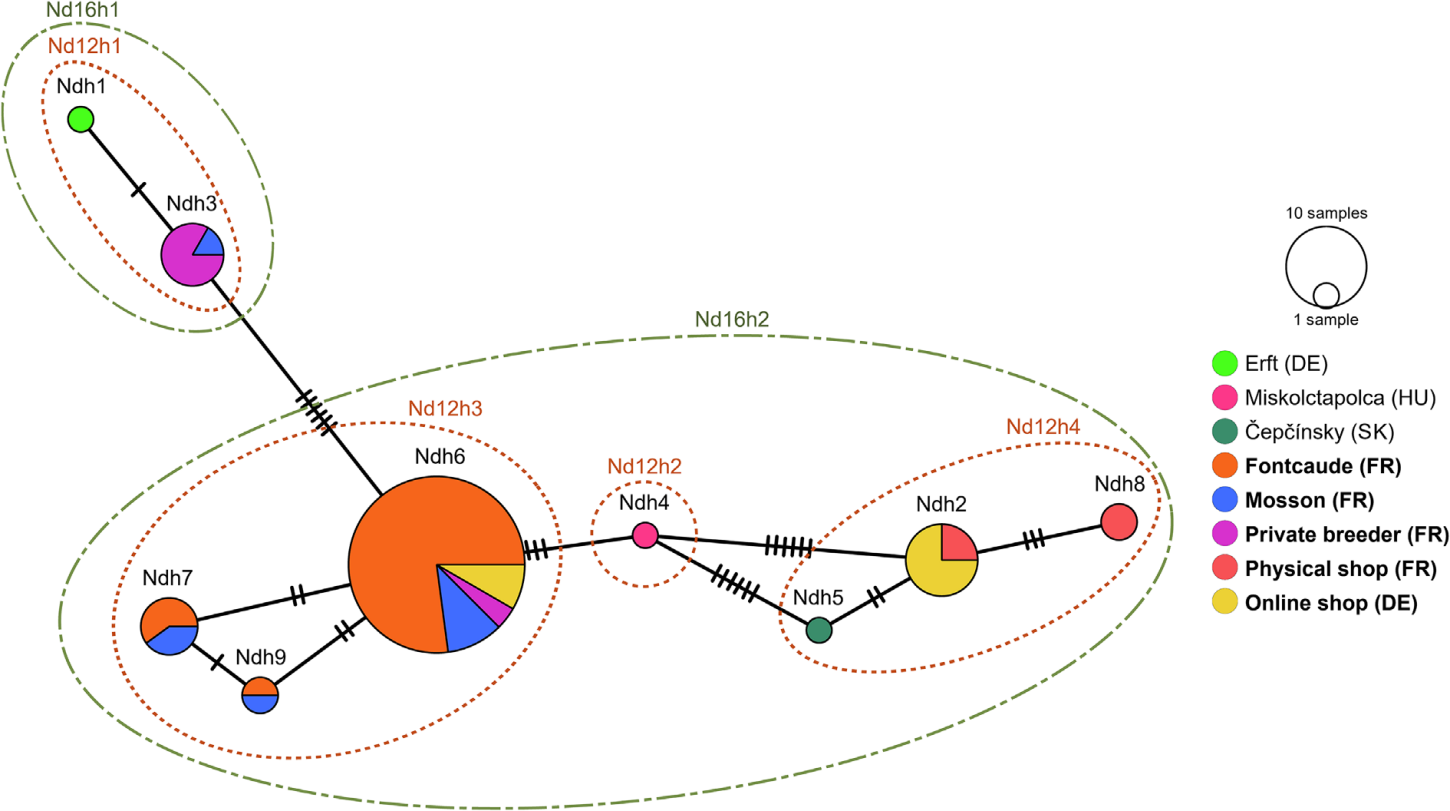
Minimum Spanning haplotype network based on COI sequences, including feral and pet traded *Neocaridina davidi* individuals obtained in this study (in bold) and revisited reference material belonging to three haplotypes (Ndh1, Ndh4, and Ndh5) from Germany, Hungary, and Slovakia, obtained in a previous study (see [52]). Dashed ellipses indicate haplotype circumscriptions based on the newly provided alternative markers 12S and 16S.

All feral and pet-traded *N. davidi* clustered in the clade B1 (Fig. 1). Feral *N. davidi* belonged to four COI haplotypes, here named “Ndh3”, “Ndh6”, “Ndh7”, and “Ndh09” following [52], and those bought from the pet trade to “Ndh2”, “Ndh3”, “Ndh6”, and “Ndh8” haplotypes (Fig. 2). The most common haplotype was “Ndh6”, which was detected in all samples except those bought from a local physical store. Likewise, “Ndh3” was shared between individuals from the Mosson and those bought from local private sellers. Haplotypes “Ndh9” and “Ndh7” were found exclusively in feral *N. davidi* individuals, the first in both the thermal park and the Mosson, and the latter only in the Mosson. Conversely, haplotypes “Ndh2” and “Ndh8” were exclusively found in pet-traded individuals; the first was shared between physical and online stores, and the latter was exclusive to a local physical store (Fig. 2).

The “Ndh2” haplotype was 100% identical to feral and pet-traded individuals from Brazil (PP718677), Canada (MG319788), Germany, Hungary, Poland, Slovakia (OR610863), the French overseas territory of La Réunion (PQ069719), and wild individuals from Taiwan (MG734262). “Ndh3” showed 100% similarity to feral and pet-traded *N. davidi* from Germany (OR610861) and wild Taiwanese specimens (MG734280), while “Ndh6” shared 100% similarity with those previously found in the Fontcaude thermal park (PP593877). The haplotypes “Ndh7”, “Ndh8”, and “Ndh9” are reported for the first time in this study (Figs. 1 and 2).

The newly developed primers successfully produced forward sequences of ~600 bp (12S) and ~1,000 bp (16S). These regions were less variable than the COI and resulted in fewer haplotypes. The obtained sequences clustered into four 12S haplotypes and two 16S haplotypes, with divergences of 0.18–0.71% and 0.01%, respectively, further confirming that all analyzed individuals belonged to a single species with a common origin (Fig. 2).

### Microsporidian identification and their phylogenetic relationships

**Figure 3:**
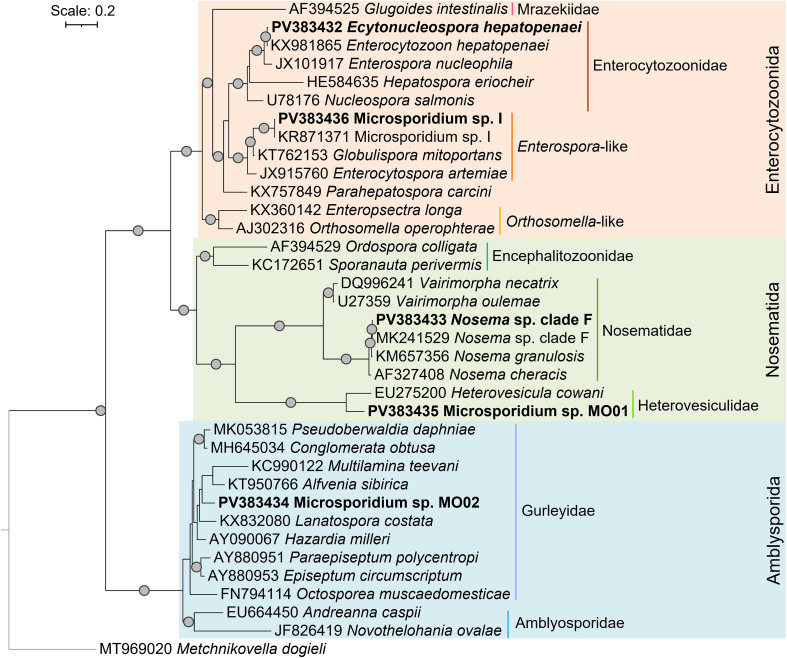
Maximum likelihood phylogenetic tree of microsporidians identified in this study. Grey dots represent bootstrap support values above 90%. Sequences obtained in this study are indicated in bold. The substitution model used was TIM3 + F + I + R4, and *Metchnikovella dogieli* represents the outgroup. The names and circumscriptions of microsporidian orders followed [5].

In total, 26 out of 320 (8.12%) *N. davidi* individuals were infected with microsporidians. With only one immature individual infected, the prevalence of microsporidians was higher in adults. Among mature individuals, microsporidian prevalence did not differ between sexes and was not influenced by host size (all ORs 95% CI crossing 1, Table 1). With 24 out of 268 (8.96%) compared to 2 out of 52 (3.85%) infected shrimp, the overall prevalence of microsporidians in feral *N. davidi* was more than double that of pet-traded individuals. However, this difference was not statistically significant (Fisher’s exact test, *p* = 0.277). While pet-traded *N. davidi* (only those from a local physical store) were exclusively infected with *E. hepatopenaei* (99.70% similarity and 100.00% coverage to KX981865), feral individuals hosted three microsporidian parasites (Fig. 3, Table 2). The most common parasite in feral *N. davidi*, with a prevalence of 5.60% (15/268), was a newly isolated microsporidian, here named Microsporidium sp. MO02, while the two remaining microsporidians, Microsporidium sp. I and *Nosema* sp. clade F *sensu* [2] reached a prevalence of 2.24% (6/268) and 1.12% (3/268), respectively. Microsporidium sp. MO02 and Microsporidium sp. I were shared with other host species, respectively attaining 4.04% (18/446) and 2.24% (10/446) prevalence among all investigated crustaceans. Microsporidium sp. MO02 was only found in adult *N. davidi* in March 2024 and shared with co-occurring *A. aquaticus*. This parasite clustered among Gurleyidae in the Amblyosporida order *sensu* [5], with the most similar isolate being *Hazardia milleri* (Hazard and Fukuda, 1974) (89.70% similarity and 99.00% coverage to AY090067), isolated from the dipteran *Culex quinquefasciatus* Say, 1823. Microsporidium sp. I was found in adult *N. davidi* in September and November 2024 and shared with co-occurring *A. aquaticus*, *G. pulex*, and *P. clarkii*. In contrast, *Nosema* sp. clade F was only found in immature and adult *N. davidi* individuals in November. Microsporidium sp. I shared 100.00% similarity and coverage to KR871371, whereas *Nosema* sp. clade F had 99.63% similarity and 94.00% coverage to MK241529; both reference sequences were isolated from the amphipod *G. pulex*. Microsporidium sp. MO01, another undescribed isolate, was detected exclusively in co-occurring *G. pulex*. This parasite clustered with the most similar isolate, *Heterovesicula cowani* (Lange *et al.*, 1995) (86.48% similarity and 100.00% coverage to EU275200), isolated from the orthopteran *Anabrus simplex* Haldeman, 1852 among the Heterovesiculidae in the Nosematida order. We found no microsporidian infection in *A. desmarestii* (Table 2).

**Table 1:** Logistic regression output table from Generalized Linear Model (GLM) showing the influence of carapax size (mm) and host sex (female/male) on microsporidian infection status (infected/uninfected). The number of observations was 292, and Nagelkerke’s *R*^2^ was 0.014.

Predictors	Odds ratio	95% CI	*p*
Carapax size	1.38	0.80 – 2.37	0.249
Host sex (M)	1.01	0.40 – 2.53	0.983

**Table 2:** Prevalence of microsporidian in host species collected between March and November 2024.

Host	Pooled sample	Month	*N*	Prevalence (%)	Microsporidian
*Asellus aquaticus*	Fontcaude Park	March	6	50.00 (3/6)	Microsporidium sp. MO02
		November	3	0.00 (0/3)	
	Mosson River	November	9	11.11 (1/9)	Microsporidium sp. I
*Atyaephyra desmarestii*	Mosson River	September	3	0.00 (0/3)	
		November	41	0.00 (0/41)	
*Gammarus pulex*	Fontcaude Park	March	4	25.00 (1/4)	Microsporidium sp. MO01
		September	24	8.33 (2/24)	Microsporidium sp. I
		November	8	0.00 (0/8)	
	Mosson River	November	17	0.00 (0/17)	
*Neocaridina davidi*	Fontcaude Park	March	85	17.65 (15/85)	Microsporidium sp. MO02
		September	62	1.61 (1/62)	Microsporidium sp. I
		November	54	0.00 (0/54)	
	Mosson River	September	8	0.00 (0/8)	
		November	59	8.47 (5/59)	Microsporidium sp. I
				5.08 (3/59)	*Nosema* sp. clade F
	Pet trade	March	37	5.41 (2/37)	*Enterocytozoon hepatopenaei*
		October	15	0.00 (0/15)	
*Procambarus clarkii*	Fontcaude Park	September	11	9.09 (1/11)	Microsporidium sp. I

## Discussion

### Current status and expansion of *N. davidi* in European temperate waters

The presence of feral *N. davidi* populations in continental Europe dates back at least to 2003, with the establishment of a population in northern Poland [22]. In the following years, additional established feral populations have been found in Germany, Hungary, and Slovakia, with individuals reported from both thermal and temperate waters, indicating ongoing expansion into colder waters [30, 52, 71]. More recently, a self-sustaining feral *N. davidi* population was reported from metropolitan France, at the location investigated in the present study, the Fontcaude Thermal Park [24]. There, the natural warm water flows from the park into the nearby Mosson River [24], where the environmentally adaptable *N. davidi* appears to have found a suitable habitat. Accordingly, juveniles and ovigerous females were collected in the Mosson during the September and November sampling campaigns, providing further evidence of the species’ expansion into European waters subject to natural thermal regimes.

The water temperature in the Mosson appears to vary between 5.7 °C in winter and 23.5 °C in summer (2008–2015, Naiades, https://naiades.eaufrance.fr), a range that falls within the 6–30 °C reported in *N. davidi*’s native habitats [30]. Previous observations of ovigerous females at temperatures below 10 °C by [52] suggest that reproduction in the Mosson might even occur during the winter. Habitat suitability predictions from species distribution models indicated a score of 0.6 (low suitability) for the Montpellier area [52], suggesting that the establishment within the local riverine systems of *N. davidi*, while unlikely, may still be possible. However, these predictions are based on clade B2, which represents northern populations of *N. davidi* living in colder waters in North China and Japan, and uses air temperature data as a proxy for water temperatures. This clade is phylogenetically and geographically distinct from clade B1, which we found in the present study. Nonetheless, individuals from clade B1, which make up the southern population of *N. davidi* inhabiting the warmer waters of Taiwan and south China, have also been detected in the colder waters of northern China and Japan [47, 52]. This suggests that both the southern (clade B1) and northern (clade B2) *N. davidi* populations may have comparable thermal tolerances.

Similarly to other non-native species [36], the presence of a temperature gradient from warm (Fontcaude thermal park) to colder water (Mosson River) might facilitate the adaptation of *N. davidi* individuals to colder temperatures, a process that is further favored by rising water temperatures, increase and duration of heat waves, and a decrease in cold spells during winter [22, 66, 67, 73]. Alternatively, a large number of *N. davidi* individuals might drift continuously into the Mosson, facilitating the survival of the population even in the absence of reproduction during the coldest months and/or under predatory pressure. Accordingly, most *N. davidi* individuals from the thermal park and the Mosson shared identical haplotypes, suggesting genetic flow between the two water bodies.

### The role of the pet trade

The presence of haplotypes shared between feral and pet-traded *N. davidi* clearly indicates a link between the availability of this species in the pet trade and aquarium-dumping practices. Accordingly, the “Ndh3” and “Ndh6” haplotypes were detected in feral and pet-traded individuals bought from local private sellers, suggesting releases of shrimps kept by local aquarists in the thermal park and the Mosson. Furthermore, both “Ndh3” and “Ndh6” haplotypes were also detected in pet-traded individuals bought from one of the largest online stores in Europe (see also [52]). Thus, it is possible that, to a certain degree, the local aquarists sourced their shrimps from online stores. Online stores have increasingly played an influential role in boosting the pet trade by often providing cheap and readily available animals [31]. One of the cheapest and most readily available shrimp species on the online market is *N. davidi,* which, in its red cherry coloration, can be sold for less than one Euro per individual, significantly lower than the 3–6 Euros usually proposed by physical stores. Since the release likelihood is highest for cheap, prolific, and widely available species [12, 37], the finding of feral *N. davidi* in the Fontcaude thermal park and the Mosson River is not surprising.

Commercially successful species, including *N. davidi,* are often mass-produced in aquaculture facilities. High densities in these facilities, coupled with inbreeding, immunosuppression, and suboptimal environmental and nutritional conditions, create an ideal environment for pathogens, such as microsporidian parasites, to proliferate [6, 40, 52, 59]. Several parasites may be lost along the supply chain, for instance, after preventive treatments, handling, and transportation. However, some may still find their way into private aquariums due to insufficient biosecurity measures and the high volume of traded animals [39, 48, 49, 52]. Therefore, with the release of non-native ornamental pets, parasites might be introduced alongside their host into novel environments.

### Microsporidian spillover

The current investigation revealed infections with the microsporidian parasite *E. hepatopenaei* in pet-traded *N. davidi* individuals bought from a local physical store. This parasite has previously been reported from pet-traded and feral *N. davidi* in continental Europe and feral individuals in the French overseas territory of La Réunion [51, 52, 56]. *Ecytonucleospora hepatopenaei*, globally recognized for causing severe economic loss in shrimp aquaculture, has a simple life cycle, is environmentally tolerant, highly contagious, and can infect a wide range of invertebrate hosts, including dragonflies and the highly invasive red swamp crayfish *P. clarkii* [10, 25, 35, 68]. This parasite is present in pet-traded *N. davidi*, suggesting that biosecurity measures implemented along the supply chain are still insufficient. Thus, infected *N. davidi* might still end up in novel environments. However, contrary to our expectations, this parasite was not detected in the feral *N. davidi* population inhabiting the thermal park or the Mosson.

The absence of any microsporidian parasite clearly originating from the pet trade in the investigated feral *N. davidi* may indicate a limited subset of introduced animals stemming from uninfected pet-traded individuals. This situation likely hindered spillover events to co-occurring crustaceans. However, it is not excluded that following ongoing aquarium dumping practices, pet-traded pathogens could end up in the investigated ecosystem in the foreseeable future.

### Microsporidian spillback

Microsporidium sp. I and *Nosema* sp. clade F were most likely the result of parasite acquisition from native species. Accordingly, Microsporidium sp. I, also referred to as Microsporidium sp. IV-B in the literature [54] is a host generalist parasite of amphipods that has been detected across Europe in *Gammarus balcanicus* Schäferna, 1923, *G. pulex*, *G. roeselii* Gervais, 1835, and *Niphargus schellenbergi* Karaman, 1932 [19, 53–55, 70]. Likewise, *Nosema* sp. clade F is a parasite found in *G. pulex* and *G. balcanicus* populations from Central and Meridional Europe, including *G. pulex* individuals collected from the same region of France where our study was conducted [2]. Moreover, several members of the genus *Nosema* are known to infect decapods, lepidopterans, neuropterans, and hemipterans [2, 64]. The presence of *Nosema* sp. clade F in feral *N. davidi,* and in the case of Microsporidium sp. I also in *A. aquaticus* and *P. clarkii,* have not previously been reported. The novel Microsporidium sp. MO02 isolate also seems to be a generalist host parasite. It was shared with *A. aquaticus* and only detected when *A. aquaticus* was present in the spring water body, suggesting local origins. Such microsporidians may be able to infect a broader range of species than initially expected, thereby providing a high potential for spillback events. Thus, this reinforces the notion that generalist parasites with a presumably simple life cycle are those that persist better in anthropized environments [53].

The presence of locally acquired host-generalist parasites in feral *N. davidi* supports our second hypothesis that parasite spillback is favored over spillover. According to the definition of parasite spillback of Kelly *et al.* [29], when a non-native species functions as an alternative host for native or previously established non-native parasites, it might amplify infection levels in native host populations. For this to happen, the non-native species must acquire native or previously established parasites, be a suitable host in which parasite populations can amplify, attain sufficiently high densities relative to native species so that it can act as a reservoir of infection, and ultimately spillback parasites to the native species [29, 63]. *Neocaridina davidi* seems to be a suitable alternative host for native or previously established parasites, possibly due to the large number of individuals thriving at the site. Host density is considered a primary driver for transmission rate [1], and several host-generalist microsporidians can switch hosts [60]. Hence, it seems profitable for native or previously established microsporidians to switch from native hosts to the abundant *N. davidi*, as this will ensure their persistence and enhance their transmission within the ecosystem. *Nosema* sp. clade F infected specimens of *N. davidi,* even when the examined native host was not infected. Although we have likely not detected these parasites in native species due to the lower sampling size compared to *N. davidi*, this finding suggests that *N. davidi* could act as a reservoir of infection for native microsporidians. Moreover, seasonal dynamics might have influenced the partitioning of parasitic infections among the hosts, as microsporidian prevalence and diversity varied between seasons, supporting our third hypothesis. Importantly, Microsporidium sp. MO01 and MO02 were only detected in March, while Microsporidium sp. I and *Nosema* sp. clade F were only detected in the fall samples. Therefore, we cannot confirm that parasite spillback has occurred locally with the available data; however, all the prerequisites are present.

## Conclusion

All in all, this study provides evidence that non-native, pet-traded species may be suitable alternative hosts for native or previously established host-generalist microsporidian parasites and possess all the prerequisites necessary for parasite spillback events to occur. If spillback events occur, they may impact native host species at both the individual and population levels. Furthermore, our data support the evidence of ongoing range expansion of feral *N. davidi* into European temperate waters, which is likely bolstered by human-mediated introductions. Therefore, with the ongoing release of pet-traded species into novel environments, particularly in those influenced by warm water, we urge the implementation of preventive measures, including stricter biosecurity measures for pet-traded organisms at all levels of the supply chain, seasonal monitoring of population dynamics in invaded environments, eradication of feral populations, enhanced recognition of responsible pet ownership, and education campaigns tailored to the general public perception of invasive species.
